# Case Report: Successful Use of Minoxidil to Promote Facial Hair Growth in an Adolescent Transgender Male

**DOI:** 10.3389/fendo.2021.725269

**Published:** 2021-09-29

**Authors:** Kenneth C. Pang, Thomas P. Nguyen, Rita Upreti

**Affiliations:** ^1^ Clinical Sciences, Murdoch Children’s Research Institute, Parkville, VIC, Australia; ^2^ Department of Adolescent Medicine, Royal Children’s Hospital, Melbourne, VIC, Australia; ^3^ Department of Paediatrics, University of Melbourne, Parkville, VIC, Australia; ^4^ Inflammation Division, Walter and Eliza Hall Institute of Medical Research, Parkville, VIC, Australia; ^5^ School of Medicine, Western Sydney University, Sydney, NSW, Australia; ^6^ Endocrinology Unit, Monash Health, Clayton, VIC, Australia; ^7^ Clinical Andrology Service, Hudson Institute of Medical Research, Clayton, VIC, Australia; ^8^ Endocrinology and Diabetes Unit, Western Health, Melbourne, VIC, Australia

**Keywords:** transgender, gender dysphoria, adolescent, minoxidil, hair growth

## Abstract

Increasing numbers of trans and gender diverse young people are presenting to health services seeking gender-affirming medical care. While testosterone therapy in transgender males is generally effective in inducing masculinization, some adolescents encounter barriers to accessing such treatment or may not wish to experience all the changes that usually accompany testosterone. Here, we describe the case of a 17 year old trans male who presented with gender dysphoria but was initially unable to start testosterone therapy. Due to a desire for facial hair, he was therefore treated with topical minoxidil, an easily accessible, over-the-counter medication that has been used to treat androgenic alopecia for several decades. In this case, minoxidil was applied regularly to the lower face and, after three months of treatment, he developed obvious pigmented facial hair that was sufficient to help him avoid being misgendered. The only reported side effect was excessive skin dryness. Unexpectedly, despite no direct application to other areas, there was also an increase in pigmented body hair, suggestive of systemic absorption and effect. Given its long-standing use and safety record in the management of alopecia, minoxidil might thus represent a useful treatment option for trans males who desire an increase in facial hair.

## Introduction

Trans and gender diverse (TGD) individuals have a gender identity that differs from their sex assigned at birth. Gender dysphoria (GD) refers to the psychological distress that may arise from this incongruence. Recent estimates suggest 1.2-2.7% of young people within the general population identify as TGD (1, 2). With a high rate of associated mental health problems observed among the pediatric TGD population (e.g. depression, anxiety, self-harm, suicidality), there is a strong impetus for health professionals to provide early and effective gender-affirming care (3–7).

TGD individuals assigned female at birth have come to represent the majority of adolescents presenting to specialist gender clinics (8, 9). The majority of these individuals have a transmasculine identity, and testosterone is frequently used to induce reversible changes such as android fat redistribution and increased muscle mass, as well as irreversible changes such as a deeper voice and increased facial/body hair growth (10, 11). The choice of medical interventions varies and is often influenced by the desire to acquire particular masculine characteristics and/or avoid certain side effects. For instance, some individuals might want to delay or avoid testosterone, but desire access to treatments that help remove unwanted breast tissue (e.g. masculinizing chest surgery) or promote facial hair growth to avoid misgendering.

Currently, however, there are no recommended treatments used to specifically stimulate facial hair development in transmasculine individuals. Indeed, a recent literature review of potential therapies for specifically encouraging facial hair growth – including in cis-gender individuals – identified only two previous clinical trials (12). One trial examined use of topical 2.5% testosterone gel applied for six months to the beard area in men with thalassemia major – since male hypogonadism is a known complication of this condition – and reported a significant increase in facial hair density compared to those who received placebo gel (13). The other was a randomized, double-blinded, placebo-controlled trial of 3% minoxidil solution involving 48 Thai men, and which demonstrated a significant increase in facial hair counts after 16 weeks of treatment (14). Consistent with this, some transmasculine adults in online forums have described use of over-the-counter topical minoxidil to augment facial hair growth in the context of pre-existing systemic testosterone therapy. The concomitant use of testosterone in these informal reports, however, makes it difficult to know whether it was minoxidil, testosterone or their combination that was responsible for apparent improvement.

Minoxidil was first approved as an oral vasodilatory anti-hypertensive in 1979 (15). Early users noted increased hair growth (16) and, by the late 1980s, topical 5% formulations were developed to treat alopecia areata. Subsequent trials in cisgender individuals demonstrated beneficial effects in androgenetic alopecia (17–20). Since 1997, topical minoxidil has been available over-the-counter and, whilst oral minoxidil has adverse cardiovascular effects, side effects of topical administration have so far been limited to reversible hypertrichosis, pruritis and local skin irritation (16, 18–20).

Given its well-established role in promoting hair growth and relatively strong safety profile, topical minoxidil may represent an attractive treatment option for some transmasculine individuals, especially those wishing to increase facial hair growth either selectively or as part of a more generalized masculinization. However, we are unaware of any reports in the medical literature describing the use and effectiveness of topical minoxidil to stimulate facial hair growth in trans males. In this case report, we describe the safe and successful application of topical minoxidil in a trans male adolescent in the absence of any testosterone treatment.

## Case Description

### Patient Information and Diagnostic Assessment

A 16 year old, previously well individual who was assigned female at birth presented to a pediatric gender clinic at the Royal Children’s Hospital in Melbourne, Australia, with a transmasculine gender identity and, upon further mental health assessment, was diagnosed with GD as per DSM-5 criteria by an experienced child and adolescent psychiatrist. Previously, he had begun questioning his gender identity with the onset of puberty around age 10 years, started binding his chest at age 11 years, and subsequently disclosed his male gender identity to his friends and parents at age 14 years. After perceiving that his parents were overwhelmed by this news, he did not raise the issue again with them until a year later, after which he requested access to gender-affirming health care. At the Royal Children’s Hospital, he was seen by a specialist multidisciplinary gender team, including a clinical nurse consultant, psychologist, psychiatrist, and pediatrician, who together helped manage his GD, which included facilitating a social transition, ensuring safe use of a chest binder, and prescribing norethisterone to suppress his menses, which had started at age 12 years and had not been previously associated with any problems (apart from gender dysphoria). However, due to legal requirements in Australia at the time, he was required to obtain Family Court approval to initiate therapy with testosterone.

### Therapeutic Intervention, Follow-Up and Outcomes

**Figure 1:**
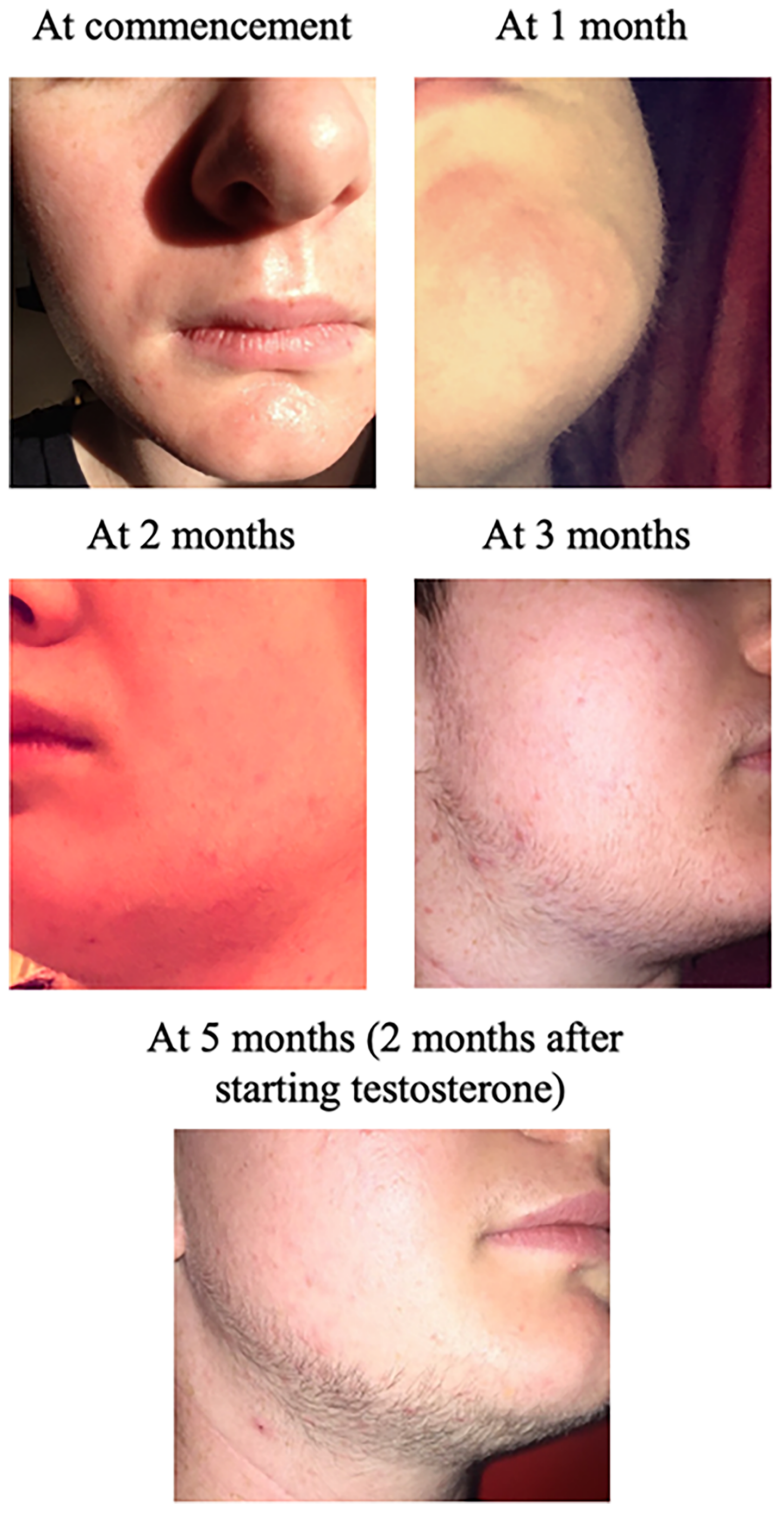
Facial hair growth following topical minoxidil treatment. Minoxidil (5% lotion, 1 mL) was applied twice daily to the beard area. Photos were taken by the patient to document changes in facial hair at the indicated time points.

While awaiting this approval and now aged 17 years, the patient asked whether he could try minoxidil to develop facial hair based on online accounts he had read. Clinical characterization at this time was unremarkable (**Table 1**) with no evidence of polycystic ovarian syndrome (e.g., no history of irregular menstrual cycles to suggest ovulatory dysfunction) nor hyperandrogenism [e.g. no signs of male pattern baldness, acne or hirsutism (see **Table 2** for modified Ferriman Gallwey score evaluation at baseline)]. With his pediatrician’s agreement, he subsequently purchased minoxidil (5% lotion) and applied 1 mL twice daily to the beard area. Shortly after commencement, he noted skin dryness and irritation, and decreased application to five days/week and began regular moisturizing. Within one month, early fine hair growth was observed and he started shaving. By three months, there was significantly more facial hair, both in terms of number, thickness and length (up to 1 cm) (**Figure 1**). Interestingly, he also noted an increase in hair in areas with no direct topical application, including the eyebrows, inner forearms, fingers, posterior calves, dorsum of feet and toes, chest and lower abdomen (**Table 2**). After three months on minoxidil, he was able to commence testosterone therapy. His subsequent medical transition, which included bilateral mastectomy at age 18, has continued to proceed well and he currently remains on testosterone therapy under the care of adult gender services. At the time of writing, he continues to intermittently apply minoxidil to aid further facial hair growth, targeting areas where he feels additional growth is needed.

**Table 1:** Patient characteristics.

**Assigned sex**	Female
**Gender identity**	Male
**Age at referral**	16 years
**Age at commencement of minoxidil**	17 years
**Past medical history**	Nil relevant
**Baseline body mass index**	24.5 kg/m^2^
Baseline testosterone*	0.7 nmol/L
Baseline estradiol*	55 pmol/L
Baseline LH*	4.5 IU/L
Baseline FSH*	5.3 IU/L
**Existing medication**	Norethisterone 10 mg twice daily

*Ideally these hormone levels – in conjunction with adrenal androgens – would have been measured in the absence of norethisterone treatment to fully gauge the underlying baseline androgenic profile, but the patient did not wish to cease norethisterone at this time.

**Table 2:** Modified Ferriman-Gallwey score evaluation before and after 3 months of minoxidil monotherapy.

Area*	Before Minoxidil	After 3 months of Minoxidil
upper lip	0	2
chin	0	3
chest	0	1
upper abdomen	0	1
lower abdomen	1	1
thighs	1	1
back	0	1
arm	1	1
buttocks	0	0
*Total score*	*3*	*11*

*The modified Ferriman-Gallwey score grades the nine listed body areas from 0 (no hair) to 4 (virile). A total score of ≥8 is considered elevated for a Caucasian individual assigned female at birth.

## Discussion

Gender affirming care for TGD youth includes social and psychological support and, in some cases, medical interventions. In trans males, there may be a desire for increased facial hair growth, since this is an archetypal masculine feature and one which often helps reduce misgendering. In this case report, we describe the use of minoxidil 5% lotion with good effect in a trans male adolescent prior to and continuing after testosterone therapy. We believe that this is the first reported use of minoxidil for this purpose in the medical literature, and the facial hair development that occurred within just three months was at least as good as what we have previously observed using testosterone over a similar time frame.

While testosterone monotherapy can encourage facial hair growth, this case report suggests that minoxidil alone may also be effective in doing so and provides a new pharmacological option for TGD individuals assigned female at birth. In particular, we can envisage minoxidil being used in several different contexts. Firstly, for transmasculine individuals on testosterone, facial hair growth may occur slowly and/or to an insufficient extent despite achieving target serum testosterone levels. In such cases, minoxidil may be a means to accelerate and/or augment facial hair growth. Secondly, for individuals desiring testosterone but yet to access it, minoxidil may enable partial masculinization and thus help with GD and reduce misgendering. Although our patient’s delay in accessing testosterone was due to legal requirements in Australia at the time, TGD adolescents worldwide commonly incur barriers to accessing gender-affirming care (due to e.g. lack of suitable services, long wait times, insufficient family support) and minoxidil might therefore provide an attractive option for such young people. Thirdly, some TGD individuals may wish to masculinize without experiencing all the effects expected with testosterone. For example, some might wish to avoid impairing their reproductive function or irreversibly deepening their voice but may wish to develop facial hair. This desire for some but not all masculinizing effects is often seen in individuals assigned female at birth who have a non-binary gender identity. At present, it is difficult to accommodate such specific needs, and minoxidil may offer a potential solution.

The mechanism of minoxidil action on hair growth is unclear but has been postulated to act *via* potassium channels (21) or by causing an influx of intracellular calcium (22). Despite its unclear mechanism, the efficacy of topical minoxidil on scalp hair growth is well established in cisgender men and women. Our patient used the 5% lotion preparation of minoxidil. Typically, the 2% solution is marketed for women and the 5% solution is marketed for men. The 5% preparation has been shown to be more effective in women (18) and men (19), although it was also accompanied by increased adverse effects such as skin irritation. Our patient found the use of ‘rest days’ and moisturizer helpful in alleviating skin dryness and irritation. Alternative minoxidil preparations less likely to cause skin irritation would have been a foam (20), which is free of propylene glycol, or a 2% solution which is typically water-based.

The observation in our case that areas of skin not contacted by minoxidil demonstrated increased hair growth was unexpected and implies systemic absorption, as has been described by some (23) but not others (17). Given the potential for systemically absorbed minoxidil to have adverse cardiovascular and fetal developmental effects (16, 24), clinicians and individuals should be aware of this possibility.

Looking ahead, several important knowledge gaps remain. Firstly, one limitation of this study is that minoxidil was used as a stand-alone treatment for only 3 months before testosterone was started. A longer duration would have provided better opportunity to assess the full potential of minoxidil monotherapy to promote facial hair growth. Consistent with this, the growth observed was relatively limited, but this is to be expected given the short time frame involved. After all, facial hair takes 4-5 years to fully develop as a result of systemic testosterone treatment in transmasculine individuals (11). Therefore, it is possible that further hair growth would have been observed should our patient have continued on minoxidil alone for a longer period. but we do not know to what extent this would have occurred nor when it would have plateaued. It is thus difficult to predict if there is a limit to the amount of facial hair growth minoxidil can stimulate and how this compares to the effects of testosterone alone. Secondly, the reversibility of minoxidil therapy was not able to be assessed in this case, since the patient subsequently started on testosterone. Previous reports suggest that the effect of minoxidil in promoting hair growth in cisgender individuals is reversible, and that its cessation should lead to regression of hair growth within 3 to 4 months (24). This likely reversibility might increase the attractiveness of minoxidil for use in TGD adolescents, especially given concerns about potential long-term regret following irreversible masculinizing changes, but it would be important to specifically assess reversibility when used in this context. Future research in trans males should look towards establishing the efficacy and safety of topical minoxidil (in its two main strengths and different preparations) using clinically meaningful endpoints for facial hair growth.

In conclusion, we present the case of a 17 year old trans male who was treated with topical minoxidil prior to commencement of testosterone therapy. His outcomes demonstrate the feasibility of using topical minoxidil to augment facial hair growth in transmasculine patients and are likely to be of interest to both other patients as well as clinicians working in this area.

## Patient Perspective

Despite some skin dryness and irritation, our patient was satisfied with the effect of topical minoxidil in promoting facial hair growth. Given the legal requirements in Australia at the time, his use of minoxidil helped him to achieve a more masculine appearance while awaiting formal approval to start testosterone therapy, and he was very pleased by this. In particular, he felt that his facial hair growth while on minoxidil for the first three months helped not only to improve his gender dysphoria by allowing him to feel more comfortable with his facial appearance but also to reduce his social anxiety by decreasing the likelihood of being misgendered by others. At the time of writing, he continues to use minoxidil intermittently, which is a testament to his ongoing satisfaction with its effects.

## Data Availability Statement

The original contributions presented in the study are included in the article. Further inquiries can be directed to the corresponding author.

## Ethics Statement

Ethical review and approval was not required for the study on human participants in accordance with the local legislation and institutional requirements. The patients/participants provided their written informed consent to participate in this study. Written informed consent was obtained from the individual(s) for the publication of any potentially identifiable images or data included in this article.

## Author Contributions

RU and TN collated resources, drafted and revised the manuscript. KP was the treating clinician, conceptualized the case report, and drafted and revised the manuscript. All authors approved the final manuscript as submitted and agree to be accountable for all aspects of the work. All authors contributed to the article and approved the submitted version.

## Funding

KP wishes to acknowledge fellowship support from the Royal Children’s Hospital Foundation and the Hugh D T Williamson Foundation Trust.

## Conflict of Interest

The authors declare that the research was conducted in the absence of any commercial or financial relationships that could be construed as a potential conflict of interest.

## Publisher’s Note

All claims expressed in this article are solely those of the authors and do not necessarily represent those of their affiliated organizations, or those of the publisher, the editors and the reviewers. Any product that may be evaluated in this article, or claim that may be made by its manufacturer, is not guaranteed or endorsed by the publisher.

## Abbreviations

TGD, trans and gender diverse; GD, gender dysphoria; DSM-5, Diagnostic and Statistical Manual of Mental Disorders, 5th Edition: DSM-5.
